# Blocking Smad2 signalling with loganin attenuates SW10 cell cycle arrest induced by TNF-α

**DOI:** 10.1371/journal.pone.0176965

**Published:** 2017-05-05

**Authors:** Gao Chao, Xiaoning Tian, Wentao Zhang, Xuehai Ou, Fei Cong, Tao Song

**Affiliations:** Department of Bone Microsurgery, Honghui Hospital, Xi'an Jiaotong University College of Medicine, Xi'an, China; University of South Alabama Mitchell Cancer Institute, UNITED STATES

## Abstract

The activity of Schwann cells (SWCs) is very important in trauma-induced nerve repair, and tumour necrosis factor-α (TNF-α) produced during tissue injury inhibits the viability of SWCs, which delays the repair of peripheral nerves. Loganin is an iridoid glycoside that has been shown to alleviate a variety of cytotoxic effects. In the current study, we evaluated the potential efficacy and the mechanism of action of loganin in TNF-α-induced cytotoxicity in SW10 cells. The experimental results indicated that loganin blocked TNF-α-mediated Smad2 activation, downregulated the expression of the G1 phase cell cycle inhibitor p15^IN4KB^, and upregulated the expression of the G1 phase cell cycle activator cyclin D1-CDK4/6, which upregulated E2F-1-dependent survivin expression and relieved TNF-α-induced apoptosis in SW10 cells. The protective effect of loganin on SWCs has potential medicinal value in the promotion of peripheral nerve repair and is significant for studies in the field of tissue regeneration.

## Introduction

The treatment of peripheral nerve injury (PNI) is a problem faced by surgeons, and the physiological process that occurs after PNI resulting from trauma is called Wallerian degeneration (WD). In nerve sections, the major presentation of WD includes axonal necrosis, the decomposition and disappearance of myelin, and nerve sheath hyperplasia [1], and the repair of PNI requires approximately one month. Nerve regeneration is generally recognised to occur at a rate of 1 mm/day (mm/d), and the functional ability of the affected limb decreases by 1% that for every 6 d that recovery from nerve injury is extended [2]. Therefore, shortening the self-repair cycle of nerves should contribute to the recovery of the functional ability of the affected limb after PNI.

Schwann cells (SWCs) play very important roles in the development, functioning, and regeneration of peripheral nerves. In addition to secreting neurotrophic factors that promote axonal regeneration, SWCs also act as supporting cells to replace the myelin sheaths of peripheral nerve cells (PNCs) to increase nerve conduction velocity after nerve injury [3]. Therefore, increased SWC viability can expedite nerve repair after PNI. At the early stage of WD, PNCs and macrophages release large amounts of tumour necrosis factor-α (TNF-α) [4, 5]. Specifically, low doses of TNF-α have been shown promote the maturation and proliferation of SWCs [6, 7], whereas high doses of TNF-α induce the apoptosis of SWCs *in vitro* [8]. Because the continuous release of TNF-α by injured tissues delays the repair of PNIs, therapeutic strategies that involve blocking the synthesis or physiological transmission of TNF-α in the affected area appear feasible for facilitating recovery from PNI.

Loganin is an iridoid glycoside extracted from the plant *Strychnos nux-vomica* Linn L; it is also distributed in the plants *Oleander Branch* and *Columelliaceae*. In traditional Chinese medicine, *nux-vomica* is used to enhance limb repair after trauma, whereas loganin is used as a central nervous system (CNS) stimulant in modern medicine. Moreover, recent studies have shown that loganin inhibits inflammation [9, 10] and protects the kidney [11, 12] and nerves [13–15]. However, the mechanism by which loganin protects nerves after PNI has not been elucidated. In this study, we used a Schwann cell line, SW10, as a model to determine, for the first time, the function and mechanism underlying the action of loganin in relieving the TNF-α-induced apoptosis of SWCs.

## Materials and methods

### Antibodies and reagents

Loganin (#36483) and recombinant mouse TNF-α (#T7539) were purchased from Sigma (St. Louis, MO, USA). All primary antibodies (Rabbit) were purchased from Cell Signaling Technology (Beverly, MA, USA) and Abcam. IRDye800CW goat anti-mouse secondary antibody and Alexa Fluor^®^ 700 goat anti-rabbit secondary antibody were purchased from LI-COR (Lincoln, NE, USA), and 4',6-diamidino-2-phenylindole (DAPI) was purchased from Beyotime (Shanghai, China).

### Cell culture

SW-10 cells (CRL-2766) were purchased from ATCC, and the cells were cultured in Dulbecco’s modified Eagle’s medium (DMEM) containing 10% foetal bovine serum (FBS, Gibco) and 1% penicillin–streptomycin antibiotics (Gibco) in 5% CO_2_ at 37°C; the medium was exchanged every 2 to 3 days.

### Cell viability assay

SW-10 cells (1×10^6^ cells) were seeded in 96-well plates. After stimulation with loganin and TNF-α, the medium was exchanged, and 10 μL of CCK-8 test solution (Dojindo) was added to each well. The absorbance of the culture solution was measured at 450 nm using a microplate reader.

### Quantitative real-time PCR

A total of 1×10^7^ SW10 cells were plated in 12-well plates. After the cells were treated with loganin, TNF-α, or Smad2 RNAi, total mRNA was extracted from the cells using TRIzol and then reverse-transcribed into cDNA using a reverse transcription reagent kit. The mRNA expression of individual genes was quantified using a QuantStudio^™^ real-time fluorescence quantitative polymerase chain reaction (qPCR) system (Thermo). The primer sequences were shown in Table 1.

**Table 1:** The primer used for real-time RT-PCR analysis.

Genes	Primer sequenece	Length.nt	Tm.°C
*TGFβ1*	Forward-5’-TGGAAACCCACAACGAAATCATAG-3’ Reverse-5’-GCTAAGGCGAAAGCCCTCA-3’.	24	60.1
		19	60.7
*TGFβR2*	Forward-5’-AAAGGTCGCTTTGCTGAGGTCTA-3’ Reverse-5’-GTCGTTCTTCACGAGGATATTGGA-3’.	23	62.7
		24	61
*TGFβR1*	Forward-5’-TTCAAACGTGCTGACATCTATGC-3’ Reverse-5’-TTCCTGTTGACTGAGTTGCGATA-3’.	23	60.5
		23	60.6
*SMAD2*	Forward-5’-CAATCGCCCATTCCCCTCTT-3’ Reverse-5’-AGTCTCTTCACAACTGGCGG-3’.	20	60.7
		20	60.5
*SMAD3*	Forward-5’-ATGGCCGGTTGCAGGTGTC-3’ Reverse-5’-GGTTCATCTGGTGGTCACTGGTTTC-3’.	19	63.8
		25	64.3
*p21cip1*	Forward-5’-TTAGCAGCGGAACAAGGAGT-3’ Reverse-5’-AGAAACGGGAACCAGGACA-3’.	20	59.9
		19	59.1
*p15ink4b*	Forward-5’-TGGTGGCTACGAATCTTCCG-3’ Reverse-5’-TCGTCGCTTGCACATCCTC-3’.	20	60.4
		19	61
*p19ink4d*	Forward-5’-CACCCTGAAGGTCCTAGTGGAG-3’ Reverse-5’-AGTGGGCAGGAGAAACAAGAAG-3’.	22	62.1
		22	61.1
*p57kip2*	Forward-5’-TCGGCTGGGACCGTTCA-3’ Reverse-5’-TGTATGGCAGCTACAGCTTGTG-3’.	17	61.2
		22	61.5
*CCND1*	Forward-5’-ATGTTCGTGGCCTCTAAGATGAAG-3’ Reverse-5’-GTGTTTGCGGATGATCTGTTTGT-3’.	24	61.3
		23	60.9
*CCNE1*	Forward-5’-CAGTTTGCGTATGTGACAGATGGA-3’ Reverse-5’-GAGAAATGATACAAGGCCGAAGC-3’.	24	62
		23	60.6
*CCNE2*	Forward-5’-GGTTCAATCCCCAGCACTACA-3’ Reverse-5’-TGGCTTCCATCCACTATCCTTG-3’.	21	60.6
		22	60.4
*CCNA2*	Forward-5’-ATGATGAGCATGTCACCGTTCC-3’ Reverse-5’-TCCATTGGATAATCAAGAGGGACC-3’.	22	61.6
		24	60.5
*CDK2*	Forward-5’-GCTAGCAGACTTTGGACTAGCCAG-3’ Reverse-5’-AGCTCGGTACCACAGGGTCA-3’.	24	63.6
		20	63.3
*CDK4*	Forward-5’-CTTTCCTGCAAAACCTTAAAG-3’ Reverse-5’-GGACTCCAGTCCTCAAGCTCTG-3’.	21	54.1
		22	62.6
*CDK6*	Forward-5’-CAATAGCAATGGCGAGATCA-3’ Reverse-5’-ACCTCGGAGAAGCTGAAACA-3’.	20	56.6
		20	59.5
*p107*	Forward-5’-AGAACCACCAAAGTTACCACGAA-3’ Reverse-5’-TCTTCAGAAGCCGTAAAGTCAGC-3’.	23	61
		23	61.4
*p130*	Forward-5’-AGAAGGGTGACTGAAGTTCGTG-3’ Reverse-5’-CAACATTGACTTGGACAGGGAAG-3’.	22	60.8
		23	60.3
*RB1*	Forward-5’-AAAGGACCGAGAAGGACCAACT-3’ Reverse-5’-CAGACAGAAGGCGTTCACAAAGT-3’.	22	61.9
		23	62.1
*E2F-1*	Forward-5’-CCCCAACTCCCTCTACCCTT-3’ Reverse-5’-CTCTCCCATCTCATATCCATCCTG-3’.	20	60.9
		24	60.2
*E2F-2*	Forward-5’-CTGGAGTGCAGTGGCCTGAT-3’ Reverse-5’-TGGCTCGTGCCTGTCATCTC-3’.	20	62.8
		20	62.8
*GAPDH*	Forward-5’-GGTGAAGGTCGGAGTCAACGG-3’ Reverse-5’-CCTGGAAGATGGTGATGGGATT-3’.	21	63.5
		22	60.4

### In-cell western blotting (ICW)

A total of 1×10^6^ SW10 cells were plated in 96-well plates. After treating the cells with loganin, TNF-α, or supernatant from 293T cells for 24 h, the cells were fixed in 4% paraformaldehyde for 15 min, permeabilised in 0.1% Triton for 15 min, and blocked in 3% BSA for 30 min. Rabbit anti-mouse cleaved-caspase-3, cleaved-caspase-8, cleaved-caspase-7, and cleaved-poly(ADP-ribose) polymerase (PARP) antibodies were separately mixed with a goat anti-mouse GAPDH antibody at a ratio of 1:1 (v/v) (titre 1:50). After the addition of 100 μL of antibody solution to each well, the plates were incubated overnight. Fluorescently labelled mouse anti-goat secondary antibodies (680 nm) and mouse anti-rabbit secondary antibodies (750 nm) were mixed at a ratio of 1:1 (v/v) and incubated with the cells for 2 h. Fluorescence images at emission wavelengths of 700 and 800 nm were obtained using laser confocal microscopy, grayscale value as shown in S1 Table.

### Packaging and transfection of lentiviruses containing interference plasmids, overexpression plasmids, and luciferase reporter plasmids

TNFR1 siRNA, GV248-Smad2 shRNA, GV248-survivin shRNA, and GV248-E2F1 shRNA interference plasmids (providing three targeting sequences; the element sequence was hU6-MCS-ubiquitin-EGFP-IRES-puromycin), GV218-Smad2, which overexpresses an ORF plasmid (the element sequence was pGC-FU-EGFP-IRES-puromycin), and a GV260-survivin promoter luciferase reporter plasmid (the element sequence was ubiquitin-MCS-luciferase-IRES-puromycin) were constructed by Shanghai Genepharma. Lenti-Easy Packaging Mix (Thermo) and the lentiviral plasmids containing the target genes were co-transfected into 293T cells (the culture medium was DMEM containing 10% FBS). After 48–72 h of transfection, the cell supernatant was collected, centrifuged, filtered, concentrated, purified, and used to directly infect SW10 cells. The transfection efficiency was assessed by Western blotting.

### Luciferase reporter assay

SW-10 cells were transiently transfected with the GV260-survivin promoter luciferase reporter plasmid and the GV260-βGal control plasmid using Lipofectamine 3000 (ThermoFisher). GloMax 20/20 Luminometer (Promega) was used to measure the luciferase activities of the GV260-survivin promoter luciferase reporter plasmid, the luciferase activity values as shown in S1 Table.

### Observation of the subcellular localisation of survivin and E2F-1 using laser confocal microscopy

Cell culture dishes (2-cm diameter) were inoculated with 1×10^4^ SW10 cells/dish. After treating the cells with loganin and TNF-α, the cells were fixed in 4% paraformaldehyde for 15 min and blocked in 3% bovine serum albumin (BSA) for 30 min. Subsequently, 100 μl of a solution of rabbit anti-mouse survivin or goat anti-mouse EFF-1 antibodies (titre 1:50) was added, and the dishes were incubated overnight. The cells were then incubated with fluorescently labelled mouse anti-goat (680 nm) or mouse anti-rabbit (750 nm) secondary antibodies for 2 h and stained with 4'-6-diamidino-2-phenylindole (DAPI) for 5 min. Fluorescence images at 680 nm, 750 nm, and 488 nm were obtained using laser confocal microscopy.

### Signalling pathway screening

SW10 cells were pre-incubated with loganin for 2 h and then stimulated with TNF-α for different periods. The cell lysates were collected and analysed according to the instructions provided by the PathScan Antibody Array Kit (Cell Signaling Technology). Fluorescence emission points on the chip were observed at 680 nm using an Odyssey imaging system (LI-COR). The grey-scale value was calculated and statistically analysed.

### Cell cycle detection

SW-10 cells were seeded into 6-well plates. After appropriate treatment, propidium iodide (PI) (Beyotime Biotechnology) was used to stain the cells for 30 min. A flow cytometer (BD FACSVerse) was used to assess the cell cycle, the cell cycle distribution ratio as shown in S1 Table.

## Results

### 1. Loganin attenuated TNF-α-induced SW10 cell apoptosis

**Fig 1:**
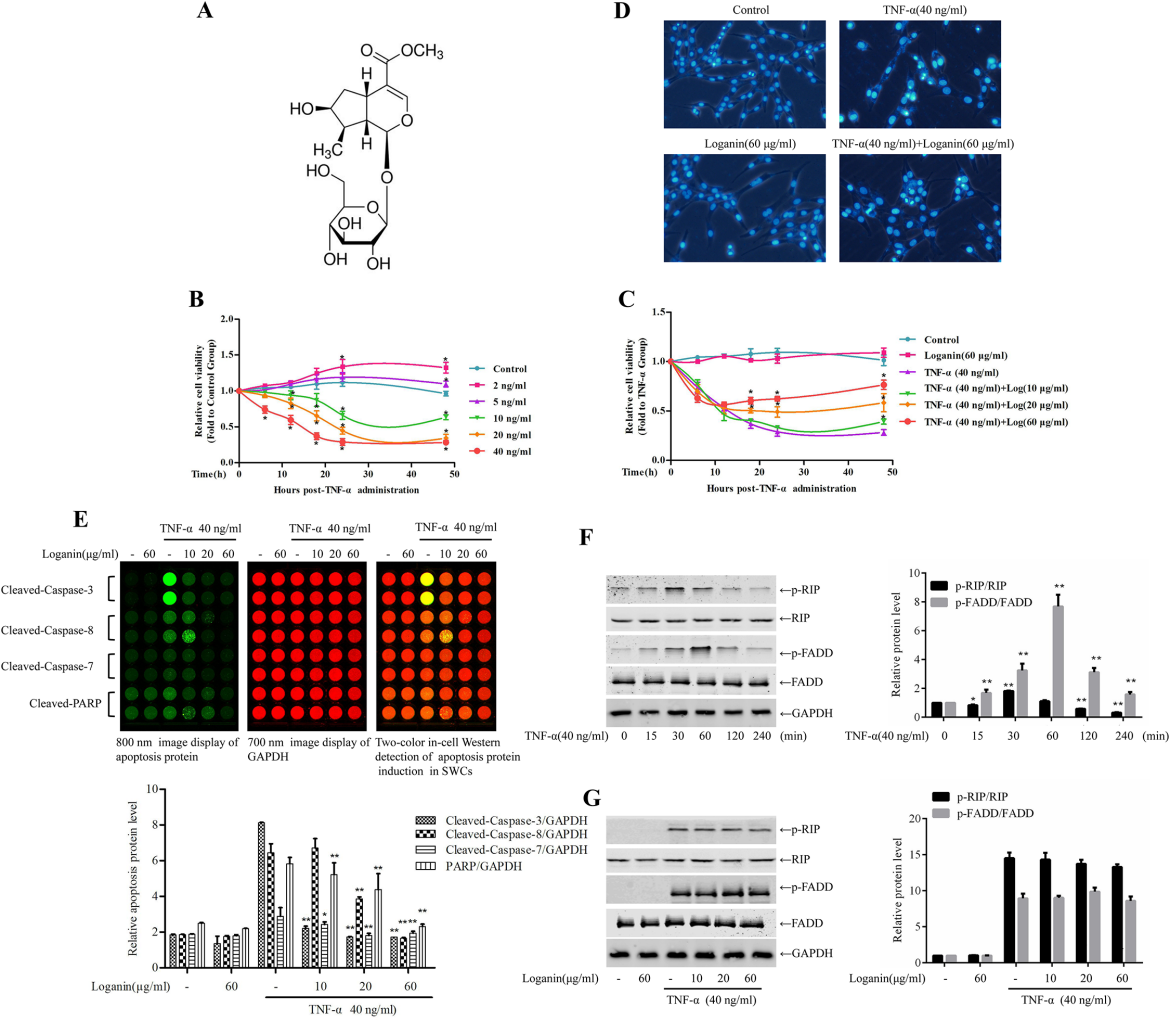
Loganin attenuated TNF-α-induced SW10 cell apoptosis. Chemical structure of loganin (A). SW10 cells were incubated with 0, 2, 5, 10, 20 and 40 ng/mL TNF-α for 6, 12, 18, 24 and 48 h, and cell viability was determined in a CCK-8 assay (B). Schwann cells were pre-incubated with 10, 20 and 60 ng/mL loganin for 2 h followed by incubation with 10, 20 and 40 ng/mL TNF-α for 24 h, and cell viability was assessed (C). SW10 cells were pre-incubated with loganin for 2 h followed by TNF-α stimulation for 24 h, and the cells were then stained with DAPI (D). The expression levels of cleaved caspase-3, cleaved caspase-8, cleaved caspase-7, and cleaved PARP were measured using the ICW method (E). SW10 cells were incubated with TNF-α for 6 h, and the phosphorylation of FADD and RIP at various time points was detected by Western blotting (F). SW10 cells were pre-incubated with loganin followed by TNF-α stimulation for 30 min or 1 h before measuring p-RIP and p-FADD (G). Data are presented as the mean±S.D, *P<0.01 vs. the Control group (B, C, n = 2). *P<0.05 and **P<0.01 vs. the TNF-α group (E, n = 3).

First, the effect of TNF-α on the viability of Schwann cells was examined. To this end, SW10 cells were incubated with 0, 2, 5, 10, 20 and 40 ng/mL TNF-α for 6, 12, 18, 24 and 48 h, and the cell viability was determined with a CCK-8 assay. As shown in Fig 1B, low doses of TNF-α promoted the viability of primary Schwann cells, whereas treatment with high doses of TNF-α inhibited the viability of Schwann cells. To test the ability of loganin to attenuate the TNF-α-induced decrease in Schwann cell viability, Schwann cells were pre-incubated with 10, 20 and 60 ng/mL loganin for 2 h, followed by incubation with 40 ng/mL TNF-α for 24 h, and the viability of the cells was then assessed. As shown in Fig 1C, loganin dose-dependently attenuated the TNF-α-induced decrease in Schwann cell viability; the EC_50_ at 6, 12, 18, 24 and 48 h was 161.71, 99.47, 21.64, 11.47, 7.32 μg/mL, respectively, indicating that the effect of concentration inversely correlated with the incubation time.

To observe the effect of loganin treatment on the nuclear morphology of SW10 cells induced by TNF-α, SW10 cells were pre-incubated with loganin for 2 h followed by TNF-α stimulation for 24 h, and the cells were then stained with DAPI. As shown in Fig 1D, TNF-α significantly induced the nuclear condensation of SW10 cells and damaged cell morphology, and pre-incubation with loganin significantly decreased TNF-α-induced cell injury. The expression of cleaved-caspase-3, cleaved-caspase-8, cleaved-caspase-7, and cleaved-PARP was measured using the ICW method. The results (Fig 1E) showed that TNF-α upregulated the expression of caspase-3, caspase-8, caspase-7, and PARP, whereas the application of loganin inhibited the expression of caspase-3, caspase-8, caspase-7, and PARP induced by TNF-α.

Fas-associating protein with death domain (FADD) and receptor-interacting protein (RIP) are 2 signalling molecules that are activated by the tumour necrosis factor receptor (TNFR)-dependent pathway. Subsequent experiments analysed the ability of loganin to block the TNF-α-induced activation of related signal transduction pathways. To this end, SW10 cells were incubated with TNF-α for 6 h, and the phosphorylation of FADD and RIP at various time points was detected by Western blotting. As shown in Fig 1F, TNF-α activated FADD and RIP in a time-dependent manner. When the cells were pre-incubated with loganin followed by TNF-α stimulation for 30 min or 1 h, loganin did not prevent the increase in phosphorylated RIP (p-RIP) and phosphorylated FADD (p-FADD)(Fig 1G), indicating that loganin does not inhibit the activation of TNFR-pathway-dependent signalling molecules. These results indicated that although loganin attenuated TNF-α-induced SW10 cytotoxicity, this effect was not mediated by blocking TNFR-dependent activation pathway.

### 2. Loganin blocked Smad2-mediated SW10 cell cycle activation

**Fig 2:**
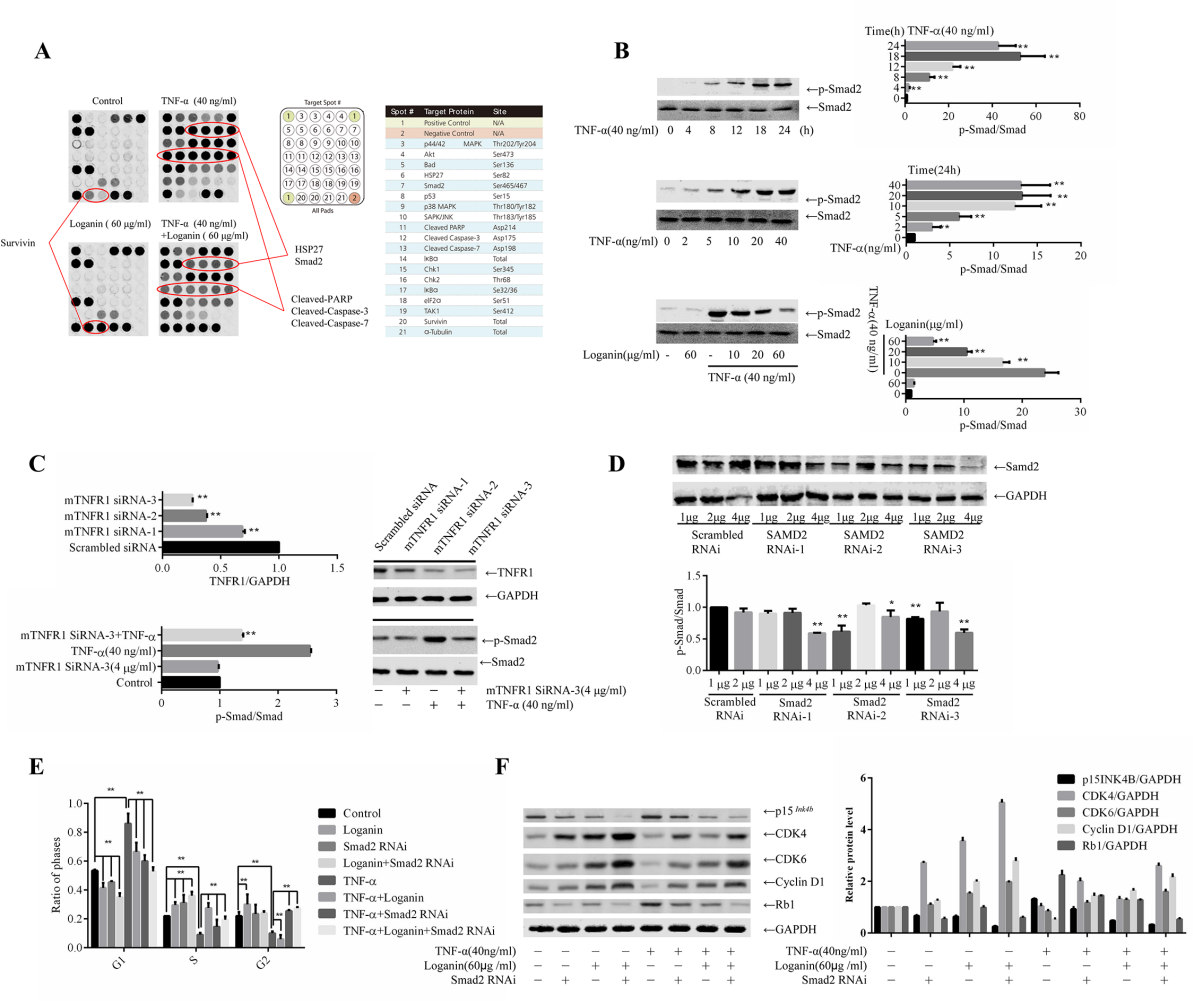
Loganin blocked SW10 cell cycle activation by Smad2. SW10 cells were incubated with loganin and TNF-α for 24 h, and changes in apoptotic signalling in SW10 cells were evaluated using a PathScan Apoptosis Signaling Antibody Array Kit (A). SW10 cells were stimulated with TNF-α for 24 h, and the level of p-Smad2 at different time points was measured; SW10 cells were stimulated with TNF-α (0, 2, 5, 10, 20, and 40 ng/mL) for 24 h; SW10 cells were pre-incubated with loganin for 2 h followed by TNF-α stimulation for 24 h, and p-Smad2 was detected by Western blotting (B). Construction of an siRNA plasmid for TNFR1 expression, transfection of SW10 cells, and Western blotting detection of interference efficiency and Smad2 activation (C). Three GV248-Smad2 shRNA plasmids targeting Smad2 expression were constructed. After 293T cells were transfected with lentiviruses carrying these plasmids, the culture supernatant from the 293T cells was collected and incubated with SW10 cells, and Smad2 expression in the SW10 cells was measured by Western blotting (D). SW10 cells were stained with propidium iodide, and the number of cells at each phase of the cell cycle was measured using flow cytometry (E). SW10 cells were incubated with loganin for 2 h or treated with GV248-Smad2 shRNA, followed by TNF-α stimulation for 24 h. The expression levels of p15^*Ink4b*^, CDK4, CDK6, cyclin D1, and retinoblastoma (Rb) protein were measured using Western blotting (F).

To investigate the mechanism underlying the ability of loganin to attenuate TNF-α-induced SW10 cytotoxicity, SW10 cells were incubated with 60 μg/mL loganin and 40 ng/mL TNF-α for 24 h, and changes in apoptotic signalling in SW10 cells were evaluated using a PathScan Apoptosis Signaling Antibody Array Kit. As shown in Fig 2A, stimulating SW10 cells with TNF-α activated apoptosis signalling molecules, including p53, JNK, p38, Smad2, and HSP27, to various degrees; in addition, caspase-3 and caspase-7 expression and PARP degradation were induced. When TNF-α action was blocked by loganin, the activation of Smad2 and HSP27 was inhibited, and the levels of caspase-3 and caspase-7 expression and PARP expression were reduced. Interestingly, incubation with loganin alone did not influence signalling in SW10 cells other than inducing high levels of survivin expression. Based on the above results, we speculated that survivin protein and Smad2 signalling play important roles in attenuating TNF-α-induced apoptosis in SWCs.

Next, SW10 cells were stimulated with TNF-α for 24 h, and the levels of phosphorylated Smad2 (p-Smad2) were measured at different time points. As shown in Fig 2B, TNF-α activated Smad2, as evidenced by the levels of p-Smad2, in a time- and dose-dependent manner, and pre-incubation with loganin inhibited this activation. Moreover, an siRNA vector for TNFR1 expression was constructed and transfected into SW10 cells, and the transfection efficiency was validated. Subsequently, Smad2 activation was assessed, which showed that blocking the expression of TNFR1 significantly inhibited TNF-α-induced Smad2 phosphorylation (Fig 2C). Furthermore, GV248-Smad2 shRNA plasmids targeting Smad2 expression were constructed, based on the results shown in Fig 2D, SMAD2 RNAi-3 was selected as the plasmid for subsequent interference. After transfection with the SMAD2 RNAi for 72 h and subsequent incubation with loganin and TNF-α for 24 h, SW10 cells were stained with propidium iodide, and the number of cells at each phase of the cell cycle was measured using flow cytometry. The results (Fig 2E) showed that TNF-α increased the number of G1-phase cells and reduced the number of S-phase cells; thus, it inhibited cell proliferation. Moreover, treating cells with loganin and Smad2 RNAi reduced the number of G1-phase cells and increased the number of S-phase cells. These results indicate that TNF-α induced cell cycle arrest. Next, SW10 cells were transfected with GV248-Smad2 shRNA followed by incubation with loganin and TNF-α for 24 h, and the levels of mRNAs encoding cell cycle-related molecules were measured using real-time qPCR. As shown in Table 2, TNF-α upregulated or downregulated the mRNA expression of various cell cycle molecules, and the incubation of cells with loganin or their treatment with GV248-Smad2 shRNA promoted the mRNA expression of cell proliferation molecules. In addition, the expression of p15^*Ink4b*^, CDK4, CDK6, cyclin D1, and retinoblastoma (Rb) proteins was detected using Western blotting. As shown in Fig 2F, TNF-α upregulated p15^*Ink4b*^ and Rb expression and downregulated CDK4, CDK6, and cyclin D1 expression compared with the control. Compared with the TNF-α group, loganin incubation and SMAD2 RNAi treatment downregulated p15^*Ink4b*^ and Rb expression and upregulated CDK4, CDK6, and cyclin D1 expression. These results indicated that loganin may have attenuated TNF-α-mediated cell cycle arrest by inhibiting the activation of Smad2 signalling.

**Table 2:** Fold-changes in specific genes in SWCs treated with TNF-α and loganin.

2^-ΔΔCt	Fold-changes to Control group			Fold-changes to TNF-α group		
	Control	TNF-α (40 ng/ml)	Loganin (60 μg/ml)	TNF-α (40 ng/ml) +Loganin (60 μg/ml)	TNF-α (40 ng/ml) +Smad2 RNAi (4μg/ml)	TNF-α (40 ng/ml) +Smad2 RNAi (4μg/ml) +Loganin (60 μg/ml)
Transforming growth factor, β 1 (TGF β 1)	1	1.46**↑	1.07	1.06	1.02	1.02
Transforming growth factor,β receptor II (TGF β RII)	1	1.01	0.98	0.98	1.04	0.99
Transforming growth factor,β receptor 1 (TGF β RI)	1	0.98	0.97	1.01	1.03	1.04
SMAD family member 2 (SMAD2)	1	4.32**↑	0.78**↓	0.55##↓	0.31##↓	0.31##↓
SMAD family member 3 (SMAD3)	1	3.37**↑	0.91*↓	0.85##↓	1.03	0.82##↓
CDK inhibitor 1A (p21 *Cip1*)	1	2.25**↑	0.96	0.33##↓	0.56##↓	0.23##↓
Cyclin-dependent kinase inhibitor 2B (p15 *Ink4b*)	1	7.72**↑	0.65**↓	0.41##↓	0.29##↓	0.18##↓
Cyclin-dependent kinase inhibitor 2D (p19 *Ink4d*)	1	2.99↑	1.03	1.02	1.02	1.02
Cyclin-dependent kinase inhibitor 1C (p57 *Kip2*)	1	3.02↑	0.94	0.97	0.97	1.02
Cyclin D 1 (CCND1)	1	0.37**↓	2.56**↑	2.47##↑	4.13##↑	5.12##↑
Cyclin E 1 (CCNE1)	1	0.55**↓	1.02	0.94	0.97	0.99
Cyclin E 2 (CCNE2)	1	0.67**↓	1.12*↑	1.05	1.01	1.03
Cyclin A 2 (CCNA2)	1	0.44**↓	1.02	1.02	0.96	0.99
Cyclin-dependent kinase 2(CDK2)	1	0.26**↓	0.94	1.04	1.09	1.06
Cyclin-dependent kinase 4(CDK4)	1	0.27**↓	3.22**↑	1.78##↑	2.17##↑	2.03##↑
Cyclin-dependent kinase 6(CDK6)	1	0.31**↓	4.78**↑	2.11##↑	1.78##↑	2.78##↑
Retinoblastoma-like 1 (p107)	1	1.32**↑	1.04	0.99	0.99	1.04
Retinoblastoma-like 2 (p130)	1	1.43**↑	0.97	0.96	0.96	0.99
Retinoblastoma 1 (RB1)	1	1.79**↑	0.68*↓	0.88##↓	0.51##↓	0.45##↓
E2F transcription factor 1 (E2F1)	1	0.37**↓	2.59**↑	4.31##↑	3.51##↑	5.15##↓
E2F transcription factor 2 (E2F2)	1	0.89*↓	1.11*↑	1.02	1.04	1.02

"*",Fold-changes to Control *p<0.5;**p<0.1

"#",Fold-changes to TNF-α #p<0.5;##p<0.1

SW10 cells were transfected with GV248-Smad2 shRNA followed by incubation with loganin and TNF-α for 24 h, and the levels of mRNAs encoding cell cycle-related molecules were measured using real-time qPCR. "*", Fold-changes compared to Control *P<0.5; **P<0.1."#", Fold-changes compared to TNF-α #P<0.5; ##P<0.1.

### 3. Survivin attenuated TNF-α-induced SW10 cell apoptosis

**Fig 3:**
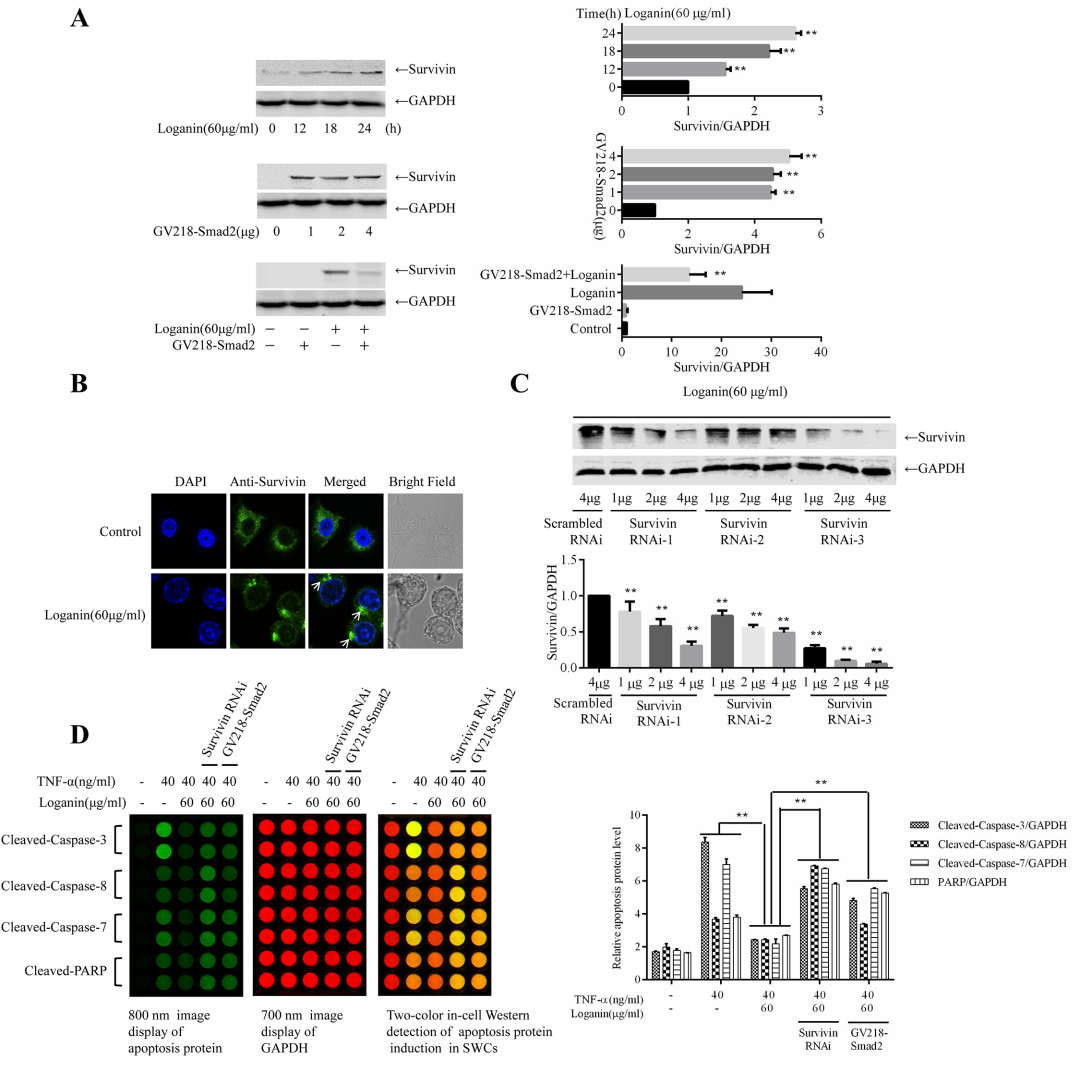
Survivin attenuated TNF-α-induced SW10 cell apoptosis. Schwann cells were incubated with 60 μg/mL loganin for 6, 12, 18, and 24 h, and survivin expression was detected by Western blotting; A SMAD2 RNAi plasmid was constructed and transfected into SW10 cells, and the survivin expression was detected by Western blotting; A GV218-Smad2-overexpressing plasmid was constructed and transfected into SW10 cells for 72 h and incubated with loganin for 24 h, the expression of survivin was measured (A). The loganin-treated cells were incubated with a green fluorescent protein-labelled survivin antibody, and the distribution of the fluorescence intensity in cells was observed using laser confocal microscopy (B). GV248-survivin shRNAs targeting 3 sites of survivin expression were constructed and transfected into 293T cells. The supernatant was collected and used to treat SW10 cells, and survivin expression was then measured (C). SW10 cells were separately exposed to survivin RNAi-3 and to GV218-Smad2-overexpressing plasmids for 72 h; the cells were then incubated with loganin and TNF-α, and the expression levels of cleaved caspase-3, cleaved caspase-8, cleaved caspase-7, and cleaved PARP were measured using ICW (D). Data are presented as the mean±S.D, *P<0.05 and **P<0.01 (F, n = 2).

Survivin has been shown to be highly expressed in actively proliferating cells, such as tumour cells and embryonic cells [16]. In the antibody array experimental results described above, survivin expression could be detected after the incubation of SW10 cells with loganin alone. Specifically, the incubation of SW10 cells with 60 μg/mL loganin for 6, 12, 18, and 24 h demonstrated that loganin induced survivin expression in a time-dependent manner, as shown in Fig 3A. After SW10 cells were transfected with 1, 2, 4 μg Smad2 RNAi plasmid for 72 h, the expression of survivin was measured. As shown in Fig 3A, blocked Smad2 expression increased the expression of survivin. To validate the association between Smad2 and survivin, the GV218-Smad2-overexpressing plasmid was used in experiments with SW10 cells. After SW10 cells were pre-incubated with 60 μg/mL loganin for 24 h, the expression of survivin was measured. As shown in Fig 3A, the overexpression of Smad2 blocked the loganin-induced expression of survivin, suggesting that loganin upregulates survivin expression by inhibiting Smad2 activation. The loganin-treated cells were incubated with a green fluorescent protein-labelled survivin antibody, and the distribution of the fluorescence intensity in cells was observed using laser confocal microscopy. The results shown in Fig 3B indicate that survivin aggregated after loganin stimulation, thereby inducing cell proliferation.

To validate the relationship between survivin and TNF-α-induced cell apoptosis, GV248-survivin shRNAs were used to treat SW10 cells, and survivin expression was then measured. Based on the results shown in Fig 3C, survivin RNAi-3 was selected as the plasmid for subsequent interference. Briefly, SW10 cells were separately exposed to the survivin RNAi-3 and the GV218-Smad2-overexpressing plasmids for 72 h; the cells were then incubated with loganin and TNF-α, and the expression of cleaved caspase-3, cleaved caspase-8, cleaved caspase-7, and cleaved PARP was measured using ICW (Fig 3D). Blocking survivin expression or the overexpression of Smad2 inhibited the anti-apoptotic effect of loganin. These results indicate that Smad2 activation downregulates survivin expression and mediates TNF-α-induced SW10 cell apoptosis.

### 4. Loganin activated E2F-1 to regulate survivin expression

**Fig 4:**
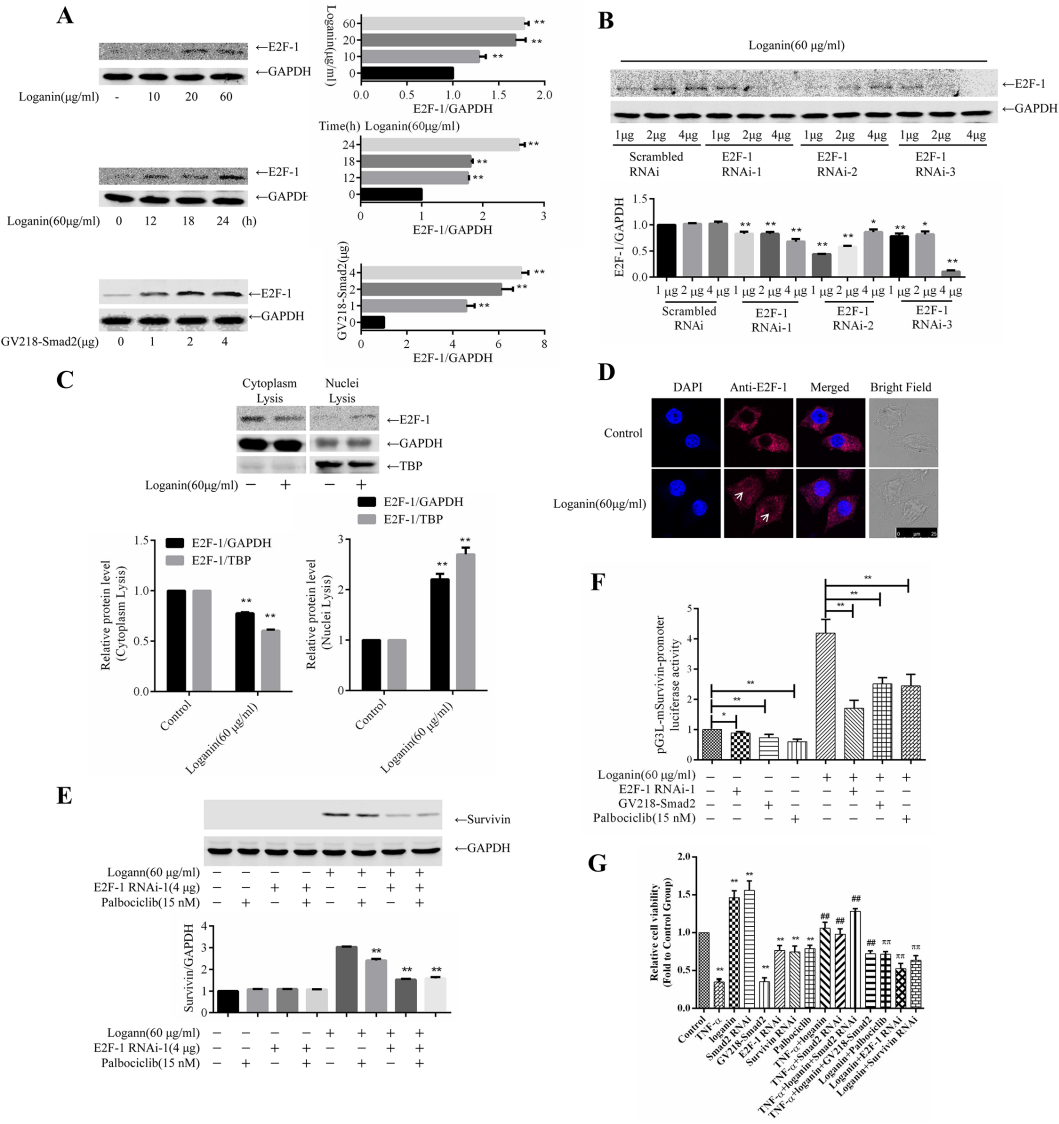
Loganin activated E2F-1 to regulate survivin expression. SW10 cells were incubated with 0, 10, 20, and 60 μg/mL loganin for 6, 12, 18, and 24 h, and the expression of the E2F-1 transcription factor was then measured; A Smad2 RNAi plasmid was constructed and transfected into SW10 cells, and the E2F-1 expression was detected by Western blotting; (A). An shRNA interference plasmid targeting E2F-1 expression was constructed and packaged into lentiviruses, and 293T cells were transfected with the plasmid. The cell culture supernatant was collected for SW10 interference, and E2F-1 was then detected by Western blotting (B). SW10 cells were treated with red fluorescent protein-labelled E2F-1 antibody, and the subcellular localisation of E2F-1 was observed using laser confocal microscopy (D). The E2F-1 RNAi-1 plasmid was transfected into SW10 cells. After 72 h, the transfected cells were incubated with loganin and the CDK4/6 inhibitor palbociclib, and the expression of survivin was measured (E). The luciferase reporter plasmid pG3L-mSurvivin was constructed and transfected into SW10 cells together with either GV218-Smad2 or E2F-1 RNAi. After the cells were incubated with loganin and palbociclib, changes in the fluorescence intensity of the cells were measured (F). Effect of blocking the SMAD2-CDK4/6-E2F-1-survivin signalling cascade on TNF-α-induced cell viability (G). Data are presented as the mean±S.D, *P<0.05 and **P<0.01 (F, n = 3). **P<0.01 vs. Control group, ##P<0.01 vs. TNF-α group, ππP<0.01 vs. loganin group (G).

The E2F-1 transcription factor family plays a critical role in cell cycle regulation [17, 18], and the qPCR results described above show that loganin can upregulate the mRNA level of E2F-1, suggesting a regulatory mechanism for survivin expression. Therefore, we evaluated the role of E2F-1 in the inhibition of TNF-α-induced cell apoptosis by loganin. To this end, SW10 cells were incubated with 0, 10, 20, and 60 μg/mL loganin for 6, 12, 18, and 24 h, and expression of the E2F-1 transcription factor was measured. As shown in Fig 4A, loganin induced the expression of E2F-1 in SW10 cells in a time- and dose-dependent manner. After SW10 cells were transfected with 1, 2, 4 μg Smad2 RNAi for 72 h, the expression of E2F-1 was measured. As shown in Fig 4A, blocked Smad2 expression increased the expression of E2F-1. After the cells were incubated with loganin for 24 h, their cytoplasmic and nuclear proteins were extracted, and the subcellular localisation of E2F-1 was determined. The results in Fig 4C show that loganin decreased the cytoplasmic E2F-1 levels and increased the nuclear E2F-1 levels, indicating that incubation with loganin induced the nuclear translocation of E2F-1. Next, the cells were treated with red fluorescent protein-labelled E2F-1 antibody, and the subcellular localisation of E2F-1 was observed using laser confocal microscopy. As shown in Fig 4D, loganin induced the nuclear translocation of E2F-1. Moreover, an shRNA interference plasmid was used for SW10 interference of targeting E2F-1 expression, and the E2F-1 RNAi-1 plasmid (Fig 4B) with the best transfection efficiency was selected for transfection of SW10 cells. After 72 h, the transfected cells were incubated with loganin and the CDK4/6 inhibitor palbociclib, and the expression of survivin was measured. The experimental results in Fig 4E show that blocking E2F-1 expression significantly downregulated loganin-induced survivin expression and that the CDK4/6 inhibitor palbociclib also inhibited survivin expression.

To confirm that loganin upregulated the activity of the survivin promoter, the luciferase reporter plasmid pG3L-mSurvivin, which contains the survivin promoter, was constructed and used for SW10 cell interference together with either GV218-Smad2 or E2F-1 RNAi. After the cells were incubated with loganin and palbociclib, changes in the fluorescence intensity of the cells were measured. As shown in Fig 4F, loganin significantly induced survivin promoter luciferase activity, whereas Smad2 overexpression, E2F-1 overexpression, and treatment with palbociclib all blocked the survivin promoter luciferase activity induced by loganin. These results indicate that the SMAD2-CDK4/6-E2F-1 signalling cascade plays a critical role in the activation of survivin expression by loganin.

Finally, we measured the effect of blocking the SMAD2-CDK4/6-E2F-1-survivin signalling cascade on TNF-α-induced cell viability. The results (Fig 4G) show that loganin and Smad2 RNAi induced cell proliferation, whereas Smad2 overexpression inhibited cell proliferation. TNF-α inhibited cell proliferation, loganin and blocking Smad2 attenuated TNF-α-mediated decreases in proliferation, and Smad2 overexpression reversed this process. In addition, blocking CDK4/6, E2F-1, and survivin all prevented the increase in cell viability induced by loganin.

## Discussion

Nerve injury is a common problem faced in the field of surgery. Currently, the major therapeutic strategies for nerve injury are autologous nerve transplantation, the application of demyelinating agents, and the use of drugs to promote PN regeneration. Moreover, increasing the activity of SWCs can also enhance the repair of nerve tissue, and the delay in the self-repair of nerves that occurs at the early stage of PNI is attributed to TNF-α. Specifically, high concentrations of TNF-α in the vicinity of injured tissue not only cause PNI but also inhibit SWC proliferation and induce apoptosis. Therefore, a therapeutic strategy that involves blocking the effect of TNF-α on SWCs to reduce the time required for nerve repair is reasonable.

Loganin is a naturally occurring small molecule that has been shown to have various cell protective functions. For example, previous studies showed that loganin can promote SW10 cell proliferation, attenuate TNF-α-mediated reductions in cell viability, and significantly decrease morphological injury in SW10 cells stimulated by TNF-α, making it a promising potential therapeutic. TNF-α exerts a dual effect: TNFR1 often mediates the activation of caspase-8 and induces apoptosis, whereas TNFR1 and TNFR2 canonically regulate NF-κB-mediated cell survival [19,20]. Protein microarray chip results showed that loganin did not block TNF-α-induced NF-κB signalling or significantly inhibit NF-κB. Further studies showed that loganin did not block the TNFR pathway-dependent FADD and RIP activation induced by TNF-α but that it downregulated the activation of apoptosis proteins induced by TNF-α. Therefore, loganin did not simply protect SW10 cells by blocking the TNF-α signalling pathway, and we consequently further explored the mechanism underlying loganin-mediated increases in SW10 cell viability.

To this end, we used protein microarrays to analyse the activation of proteins related to apoptotic signalling induced by TNF-α in SW10 cells, which showed that TNF-α activates a variety of stress molecules, including MAPKs (p44/42, p38, and JNK), Hsp27, Smad2, p53, Chk_1/2_, and NF-κB, and induces the expression of caspase-3, caspase-7, and PARP in SW10 cells. Moreover, loganin inhibited the activation of Hsp27 and Smad2 and downregulated the expression of caspase-3, caspase-7, and PARP, demonstrating that loganin interfered with the function of TNF-α. In addition, incubation with loganin alone activated a small-molecule caspase-3 inhibitory protein, survivin. Therefore, Smad2 and survivin might be target proteins through which loganin promotes SW10 cell proliferation. Western blotting showed that TNF-α persistently phosphorylates Smad2, whereas loganin inhibited the phosphorylation of Smad2 and induced survivin expression. Moreover, the transfection of cells with a Smad2 interference plasmid induced survivin expression, whereas transfection with a Smad2 overexpression plasmid inhibited survivin expression. These results indicated that Smad2 negatively regulates survivin expression. As expected, the overexpression of Smad2 and blocking survivin expression decreased the viability of SW10 cells and induced apoptosis, whereas blocking Smad2 expression increased cell viability and attenuated the reduction in cell viability induced by TNF-α. Therefore, we subsequently analysed the mechanism by which Smad2 inhibited survivin expression.

TGF-β/Smad signalling plays critical roles in the regulation of cell growth and differentiation. In many terminally differentiated tissues and cells, TGF-β/Smad inhibits excessive cell proliferation [21, 22]. Accordingly, TGF-β/Smad activity is low in embryonic cells [23] and tumour cells [24]. Because SW10 is a SV40-immortalised cell line, Smad2 activity is not detected in SW10 cells. Mutations in proteins involved in TGF-β/Smad signalling increase the risk of oncogenic effects, and mutations that affect TGF-β/Smad signalling are consequently not beneficial. However, in the field of nerve tissue regeneration, technologies that transiently inactivate TGF-β/Smad signalling in cells and thereby render terminally differentiated cells that can proliferate is very helpful for tissue regeneration. The flow cytometry results obtained in this study showed that TNF-α increased the number of SW10 cells in the G1 phase and reduced the number of cells in the S and G2 phases. Moreover, blocking Smad2 expression decreased the number of G1-phase cells, and the function of loganin was similar to that of Smad2 RNAi. These results indicate that Smad2 promotes cell proliferation and suggest that Smad2 is a functional target of loganin. In canonical TGF-β-dependent Smad signalling, G1/S transition arrest and the inhibition of cyclin-CDK signalling are mediated by p15^*IN4KB*^ and p21^*CIP1*^ expression [25–27]. Our study showed that TNF-α upregulated TGF-β1 and Smad2/3 mRNA, downregulated cyclins (cyclin A2 and E2) and cyclin-dependent kinases (CDK2, 4, and 6), and upregulated the mRNA levels of cyclin-dependent kinase inhibitors (p21^*Cip1*^, p15^*Ink4b*^, p19^*Ink4d*^, and p57^*kip2*^) in SW10 cells. Conversely, the treatment of cells with loganin downregulated Smad2/3 and p15^*Ink4b*^ and upregulated the mRNA and protein levels of cyclin D1 and CDK4/6. More importantly, Smad2 RNAi effectively blocked the increases in the expression of proteins involved in the Smad-p15^*Ink4b*^-cyclin D1-CDK4/6 signalling cascade. This phenomenon (loganin-mediated simulation of Smad2 function) indicated that loganin blocks TNF-α-induced cell cycle arrest.

The E2F transcription factor is expressed during the G1 phase of the cell cycle [28–30]. When cells are in the quiescent phase, E2F-1 is inhibited by Rb; during the G1/S transition, phosphorylated Rb dissociates from E2F-1 to activate E2F-1, and E2F-1 can then initiate the expression of a series of cell proliferation-related proteins, including survivin [31]. Our results showed that TNF-α downregulated the mRNA levels of E2F-1 and E2F-2 and their inhibitor Rb in SW10 cells, whereas loganin treatment reversed the downregulation of E2F-1 mRNA and the upregulation of Rb mRNA and protein levels. Moreover, treating SW10 cells with loganin alone activated E2F-1 in a time- and dose-dependent manner, downregulated the cytoplasmic E2F-1 levels, upregulated the nuclear E2F-1 levels, and caused E2F-1 to translocate into the nucleus and bind to survivin to initiate transcription. Moreover, blocking CDK4/6 and E2F-1 expression downregulated the transcriptional activity of the survivin promoter and the survivin protein level, whereas the overexpression of Smad2 downregulated the transcription activity of the survivin promoter. Taken together, these data indicate that loganin inhibits Smad2 signalling, activates the p15^*Ink4b*^-cyclin D1-CDK4/6 cascade, promotes E2F-1-dependent survivin expression, and upregulates SW10 cell viability to reduce SW10 cell apoptosis.

## Conclusion

**Fig 5:**
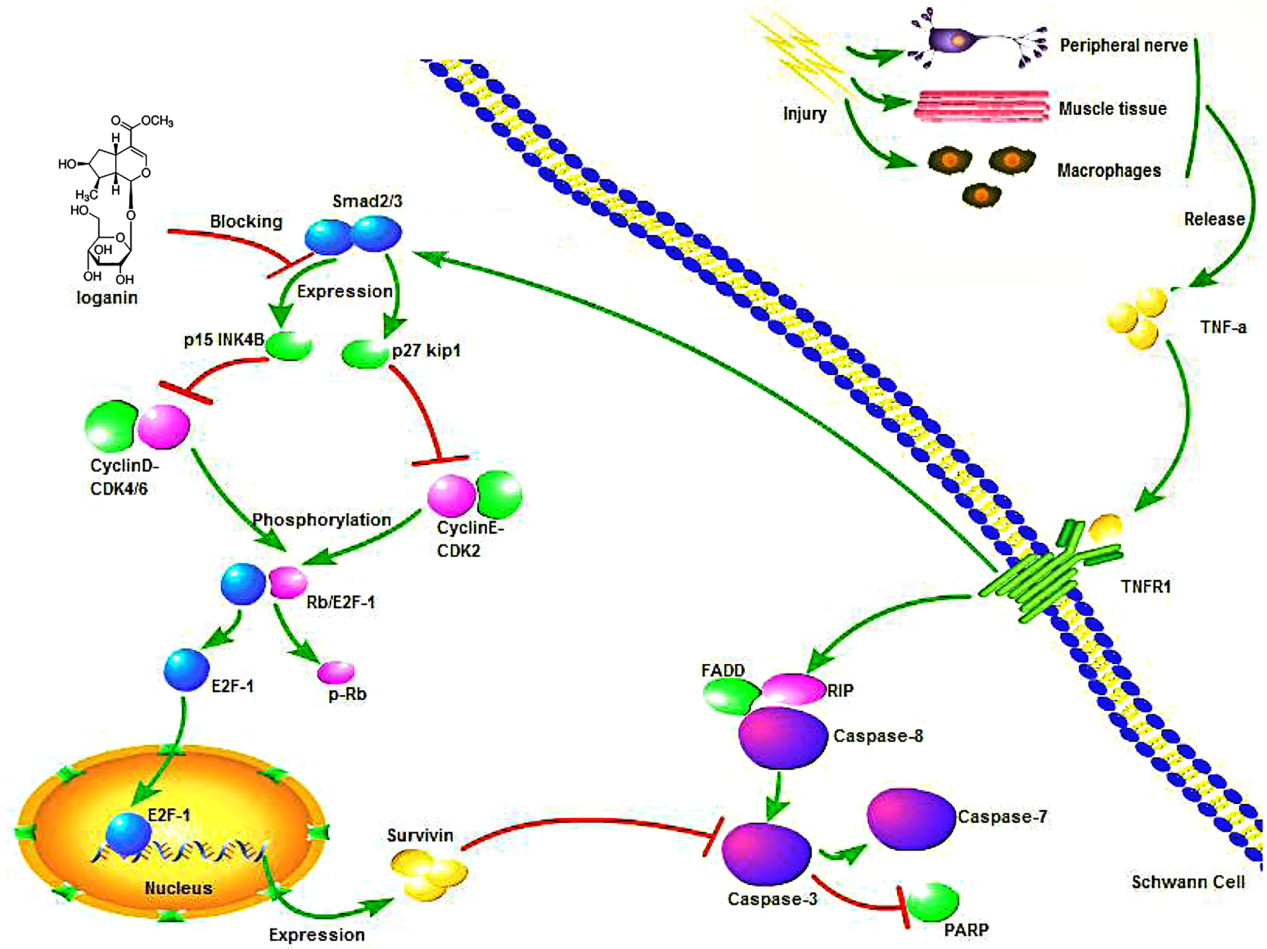
Mechanism by which loganin attenuates TNF-α-induced SWC cytotoxicity. Loganin blocks TNF-α-induced Smad2 signalling activation, activates the p15^*Ink4b*^-cyclin D1-CDK4/6 pathway, and promotes EF2-1-dependent survivin expression to attenuate reductions in SW10 cell viability and SW10 cell apoptosis due to TNF-α released by inured tissue.

In the current study, we showed that loganin blocks TNF-α-induced Smad2 signalling activation, activates the p15^*Ink4b*^-cyclin D1-CDK4/6 pathway, and promotes EF2-1-dependent survivin expression to attenuate reductions in SW10 cell viability and SW10 cell apoptosis due to TNF-α released by injured tissue (As shown in Fig 5). The inhibitory effect of loganin on Smad2 activity has potential medicinal value in the promotion of peripheral nerve repair and is significant for studies in the field of tissue regeneration.

## Supporting information

S1 Table Original experimental data. (XLSX)
